# Dissociating Affective Salience and Valence in (Very) Long‐Latency ERPs

**DOI:** 10.1111/psyp.70030

**Published:** 2025-03-11

**Authors:** Hans Revers, Jeroen J. Stekelenburg, Jean Vroomen, Katrijn Van Deun, Marcel Bastiaansen

**Affiliations:** ^1^ Department of Cognitive Neuropsychology Tilburg University Tilburg the Netherlands; ^2^ Department of Methodology & Statistics Tilburg University Tilburg the Netherlands; ^3^ Academy for Leisure & Events Breda University of Applied Sciences Breda the Netherlands

**Keywords:** allostasis, EEG, electroencephalogram, emotion, late positive potential, LPP

## Abstract

While affective salience effects have been observed consistently in the late positive potential (LPP), no event‐related potential (ERP) component has consistently shown ordered valence effects. A recent study, showing images of facial attractiveness, however suggests the existence of valence‐related effects at very long latencies (1000–3000 ms post stimulus). This could offer new insights into the time‐course of affective neural processing. Yet, it remains unclear whether the long‐latency effect was specific to facial attractiveness, or to valence in general. To corroborate the existence of a long‐latency valence effect, we presented distinctly positive, neutral, and negative valenced IAPS images to a large sample of 224 participants while recording their electroencephalogram (EEG). Larger ERP amplitudes were elicited by both positively and negatively valenced compared to neutral stimuli (an affective salience effect) from roughly 500 until 1300 ms, followed by an ordered valence effect of larger amplitudes to negatively compared to positively valenced images from 1500 until 2500 ms. These findings corroborate the previously observed sequence of an affective salience effect followed by a long‐latency valence effect. However, the polarity of this valence effect was reversed from that of the facial attractiveness study. Allostasis is discussed as potential reconciling factor. Effects in the N2 and P300 components were also found, but could not be clearly labeled as an affective salience or a valence effect. These results fit with two‐stage emotion theories such as the theory of constructed emotions.

## Impact statement

To date, no ERP component has consistently demonstrated ordered valence effects. A recent facial attractiveness study identified valence‐related responses at less commonly explored latencies (⟩1000 ms). We replicated this effect using images commonly used to elicit valence, revealing an effective salience effect in the Late Positive Potential (LPP) range, followed by a structured valence effect in the 1500‐2500 ms window. These results provide new insights into the temporal dynamics of emotional processing.

## 1 Introduction

At the heart of emotions, there are two elemental dimensions: valence (pleasant vs. unpleasant) and arousal (feeling calm vs. energized), collectively referred to as core affect (Russell 2003; Russell and Barrett 1999). The time course of affect‐related neural processing is typically studied with electroencephalogram (EEG) experiments because of its excellent temporal resolution. Many studies have shown that the late positive potential (LPP) component of the event‐related potential (ERP) is usually larger for both positively and negatively valenced stimuli compared to neutral ones (for reviews, see Hajcak et al. 2011; Olofsson et al. 2008), thus showing that ERPs are sensitive to affective salience. Affective salience is here defined as the valence distance from neutral, in either the positive or negative direction. Importantly, ERPs have failed to consistently differentiate between positively and negatively valenced stimuli (Hajcak et al. 2011; Olofsson et al. 2008). The absence of clear and robust valence effects in ERP studies limits the applicability of ERPs to study affective processes.

In a recent study, however, both affective salience and valence effects were clearly observed for images of attractive versus unattractive faces (Revers et al. 2023). Of particular interest is that in that study, a valence effect was observed at very long latencies (1000–3000 ms), an interval that is rarely explored in affect‐related ERP studies. Since demonstrating the robust existence of (very) long‐latency effects of valence in ERPs could potentially advance the research field of affective neuroscience, we aim to corroborate that these findings can be generalized to pictures of the widely used international affective picture system (IAPS, Lang et al. 2008), a standardized emotion elicitation database.

### 1.1 Valence and Affective Salience Effects in Early ERP Components

In a valence effect, ERP responses are ordered according to valence: amplitudes to positively valenced stimuli are larger than those to neutral stimuli, which in turn are larger than those to negatively valenced stimuli (positive > neutral > negative), or vice versa. Valence effects have commonly been explored in early ERP components (< 300 ms) such as the P1, N1, P2, N2, early posterior negativity (EPN), and P300 in accordance with claims from the theory of basic emotions by Ekman (1999). This theory states that emotions have a rapid onset and are of short duration (Ekman 1992), and that appraisal is automatic and must be able to function with “incredible speed” (Ekman and Cordaro 2011). However, reports of valence effects in these early ERP components are rare and inconsistent (Hajcak et al. 2011; Olofsson et al. 2008).

Affective salience effects in early ERP components, on the other hand, have been reported more consistently (Olofsson et al. 2008). Hajcak et al. (2011) note that many ERP components are sensitive to both positively and negatively valenced stimuli compared to neutral stimuli, including the P1, N1, P2, N2, EPN, and P300.

### 1.2 Affective Salience Effect in the LPP

The LPP is a positive‐going component that starts at around 400 ms, peaks at around 600–1000 ms, and can be sustained for as much as six seconds (Cuthbert et al. 2000; Yen et al. 2010). The LPP is a neural response that is thought to indicate the detection of significance in the environment (Bradley 2009; Hajcak and Foti 2020). Emotional content is intrinsically significant (Schupp et al. 2006), as it indicates a potential threat or opportunity for survival (Hajcak and Foti 2020; Öhman et al. 2001), making it motivationally relevant (Codispoti et al. 2007; Lang and Bradley 2010). The LPP has also been associated with attention and (emotional) memory (Fields 2023).

Amplitudes of the LPP component are commonly and consistently reported to be larger for affectively valenced (i.e., positively or negatively valenced) compared to neutral stimuli (Hajcak et al. 2010, 2011; Olofsson et al. 2008). In the literature, this has been labeled as an affective salience effect (e.g., Biggs et al. 2012; Niu et al. 2012) or an emotional salience effect (e.g., Hajcak and Nieuwenhuis 2006).

Affective salience refers to the degree to which something stands out compared to its surroundings as a result of the affective (or emotional) load of a stimulus. It has often been operationalized as a dichotomous variable that is either ‘affective’ (positively or negatively valenced) or ‘neutral’ (e.g., Chiu et al. 2008; Schweizer et al. 2019). In the current study, we use an operationalization that retains more of the affective information, resulting in data that are more sensitive in statistical analysis: affective salience ratings are calculated as the absolute distance of the valence rating compared to neutral.

Affective salience should not be equated with arousal. Although in some stimulus sets, normative ratings of valence appear to have a v‐shaped relationship with those of arousal: i.e. arousal increases linearly with the valence distance in both the positive and negative direction (Haj‐Ali et al. 2020; Kron et al. 2015), there is much variation between people, circumstances (Kuppens et al. 2013), and cultures (Yik et al. 2023), indicative of a complex relationship. For instance, individuals appear to differ in the degree to which they focus on valence or arousal in constructing their conscious affective experience (Barrett 1998). Some people may focus more on the valence dimension while others focus more on the arousal dimension of a subjective experience.

### 1.3 The Affective Salience—Valence Sequence Hypothesis

Using simultaneous EEG and fMRI recordings, Liu et al. (2012) showed that the affective salience effect in the LPP correlates with a pattern of neural activity that corresponds to the overlap between the emotional network (Lindquist et al. 2016) and the salience network (Schimmelpfennig et al. 2023; Seeley 2019). This has since been labeled as the affective salience network (e.g., Do et al. 2022; Metzger et al. 2023). Indeed, fMRI studies show that the affective salience effect is observable as greater BOLD responses in regions of the affective salience network (Metzger et al. 2023; Schweizer et al. 2019) consisting of the dorsal anterior cingulate cortex, the ventral‐medial prefrontal cortex, the amygdala, and the anterior insula (Metzger et al. 2023).

The anterior insula is associated with interoception (Craig 2009): internal sensations from the body that provide information on the status of resources. Affectively salient events are potentially consequential for allostasis (the efficient control of resources for all physiological systems that are needed for growth, survival and reproduction), and thus are worth the energy expenditure of further processing, while other events are safely ignored (Barrett 2017b). The human brain is an energy‐expensive organ that must work efficiently to reduce energy costs (Padamsey and Rochefort 2023). Evolution has shaped it to efficiently control resources for all physiological systems that are needed for growth, survival, and reproduction (Barrett 2017b).

Based on the two‐factor theory by Schachter and Singer (1962), MacCormack and Lindquist (2017) proposed that the evaluation of the consequences for allostasis leads to neural representations of bodily changes. These representations contribute to affective states, and eventually to emotions. We suggest that a neural processing sequence of affective salience first, followed by that of valence, would be compatible with emotion theories that posit distinct stages in emotion processing (MacCormack and Lindquist 2017) like the two‐factor theory of emotion (Schachter and Singer 1962), the James‐Lange theory (Dewey 1894; James 1884; Lange 1885), and the theory of constructed emotions (Barrett 2017a).

### 1.4 Valence Effects at (Very) Long Latencies

Since affective ERP studies have (predominantly) limited their analysis to the 1000 ms interval following stimulus presentation (Olofsson et al. 2008), valence effects at long latencies are rarely reported. Those who have reported on affective responses at long latencies (> 1000 ms) observed larger amplitudes to both positively and negatively valenced stimuli compared to neutral stimuli (Cuthbert et al. 2000; Yen et al. 2010). However, recently, Revers et al. (2023) did observe late valence effects associated with facial attractiveness. In that study, the commonly reported affective salience effect in the LPP interval (450–850 ms) was followed by a long‐latency valence effect (1000–3000 ms) with larger amplitudes to positively valenced images compared to negatively valenced images, with amplitudes to neutral images in between. Considering the clear difference in ERP amplitude between valence conditions and the long duration of the observed effect, it might be sufficiently robust to yield new insights into the timeline of emotional valence processing.

Late effects of valence could be inferred tentatively only since the pleasant, unpleasant, and neutral stimuli in that study were images of subjectively perceived attractive, unattractive, and intermediate attractive faces. The processing of faces (and facial attractiveness) is a swift and automatic, evolution‐based response (Olson and Marshuetz 2005) in brain circuits that are dedicated to face processing (Kanwisher and Yovel 2006). This makes it difficult to generalize the effects elicited by facial attractiveness to more general affect processing.

Additionally, the long‐latency valence effect in the attractiveness study was present for faces of the preferred gender only, not for faces of the non‐preferred gender. It was speculated that the differential valence response was dependent on the self‐relevance of the stimuli.

We aim to verify the existence of a (very) long‐latency valence effect in ERPs with the current study, employing a more thorough and generalizable approach. First, we use images from the widely used IAPS database (Lang et al. 2008) as stimulus material. These images are widely recognized for reliably eliciting consistent valence and arousal effects. They allow for a mixture of affective content, varying in scene complexity and evolutionary relevance, thus allowing us to establish the existence of late valence effects in more general settings. Second, since long‐latency valence effects remain largely unexplored (with only one previous finding), we will ensure robust results by testing a significantly larger number of participants than is typical in EEG studies.

### 1.5 Current Study

We selected 150 images from the IAPS database (Lang et al. 2008) in three equal‐sized categories that were clearly distinct in their normative valence ratings (positive, neutral, negative) with a broad, largely overlapping range of arousal ratings, and showed these to 224 participants while recording their EEG. Our experimental design allowed for the analysis of ERPs in a long‐latency range [0–2500 ms] to be able to detect differential responses in the long‐latency interval.

On the basis of the Revers et al. (2023) study, we expected to observe a sequential affective salience–valence effect. Specifically, we expected larger ERP amplitudes to the positively and negatively valenced images compared to the neutral images in the LPP interval (the affective salience effect), followed by an interval of larger amplitudes to positively valenced images compared to negatively valenced images (a valence effect).

## 2 Method

### 2.1 Participants

A total of 228 healthy participants (52 male, 171 female, 5 nonbinary/undisclosed; mean age = 21.0, SD = 3.5, range = 16–53 years) took part in the study after having given written informed consent. Participants were mostly undergraduate psychology students who signed up through the university's experiment participation system and received course credits. Data from four participants were excluded from analyses for having artifacts in more than half of the trials in one of the conditions. The remaining 224 participants (51 male, 168 female, 5 nonbinary/undisclosed) were 21.0 years old (SD = 3.5, range = 16–53 years), on average.

All experimental procedures were approved by the Ethics Review Board of the School of Social and Behavioral Sciences of Tilburg University (EC‐2016.48).

### 2.2 Stimuli

During the experiment, 150 IAPS (Lang et al. 2008) images were presented in color against a black background on a 24.5‐in. BenQ Zowie XL2540 LCD screen with a resolution of 1920 × 1080 pixels and a refresh rate of 240 Hz. The images were 1024 pixels wide and 768 pixels high, making them 289 by 217 mm on screen.

These images were carefully selected from the IAPS database to create 3 clearly distinct valence categories (positive/neutral/negative) based on their normative valence ratings (Lang et al. 2008). Additionally, they were selected to cover a broad range in normative arousal ratings within each valence category, yet be very similar between categories, to minimize the arousal effect. See Table 1 for mean normative valence and arousal ratings per category, and see Table A1 in Appendix A for a list of all images shown in the experiment with IAPS numbers and normative ratings.

**TABLE 1:** Mean normative valence and arousal ratings per category.

Category	Normative valence mean (SD)	Normative arousal mean (SD)
Positive	7.49 (0.36)	5.37 (1.05)
Neutral	4.96 (0.39)	4.99 (0.87)
Negative	2.49 (0.39)	5.48 (1.04)

*Note:* Normative valence and arousal ratings, as reported in the technical manual of the International Affective Picture System (IAPS; Lang et al. 2008), are averaged over the 50 images per valence category.

The similarity of low‐level perceptual features of the stimuli across positive, neutral, and negative valence conditions was assessed using MATLAB (v. R2023b, MathWorks Inc.). We converted each image to the perceptually realistic CIELAB color space (ISO/CIE, 2019) and extracted relevant low‐level features. The luminance of the images differed between conditions, *F*(2, 147) = 5.19, *p* = 0.007. Images in the positive valence condition were higher in luminance than images in the neutral, *t*(98) = 2.45, *p* = 0.016, or negative valence condition, *t*(98) = 3.25, *p* = 0.002, but did not differ significantly between the neutral and negative valence conditions, *t*(98) = 0.56, *p* = 0.58. The contrast of the images was similar between conditions, *F*(2, 147) = 1.24, *p* = 0.29. Images did not differ significantly between conditions in their green‐red balance, *F*(2, 147) = 0.90, *p* = 0.41, or in their blue‐yellow balance, *F*(2, 147) = 0.52, *p* = 0.60.

### 2.3 Design

**FIGURE 1:**
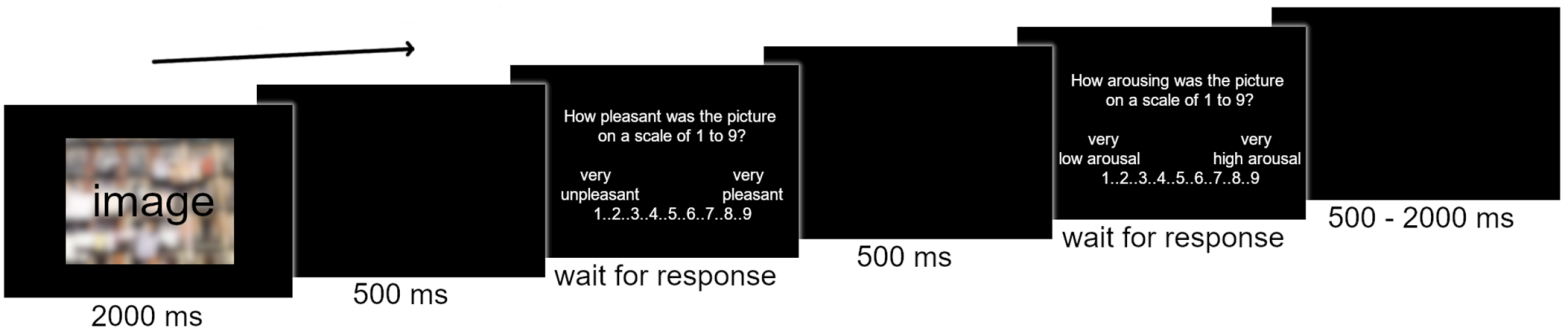
Graphical representation of a trial sequence. The sequence of a single trial: The stimulus (IAPS image) was presented for 2000 ms, followed by a blank delay of 500 ms, the question to provide a valence rating through numeric key press, another blank delay of 500 ms, the question to provide an arousal rating through numeric key press, and finally a black screen of variable duration (500–2000 ms). The image was blurred for this publication only; images were not blurred in the experiment.

Each image was presented for 2000 ms, followed first by a 500 ms blank screen, and then the question to provide a subjective valence rating on a 9‐point Likert scale (Likert 1932): “How pleasant was the picture on a scale of 1 to 9?”, with 1 marked as “very unpleasant” and 9 as “very pleasant”. Ratings were entered through a numeric key press. This was followed by another 500 ms blank screen, and subsequently the question to provide a subjective arousal rating: “How arousing was the picture on a scale of 1 to 9?”, with 1 marked as “very low arousal” and 9 as “very high arousal”. The trial ended with a black screen of variable duration (500–2000 ms) (Figure 1).

Each participant viewed all 150 images in a free viewing, within‐subjects design. Presentation order was randomized for each participant. Each image was shown only once. The 500‐ms blank screen following each image ensured a delayed response, preventing (pre)motor responses during stimulus presentation.

Valence categories (positive, neutral, negative) were used as independent variables. EEG amplitudes of the 2500‐ms interval following stimulus onset, averaged over all trials within a category, were used as dependent variables.

### 2.4 Procedure

After reading information regarding the experiment and giving written informed consent in accordance with the Declaration of Helsinki, the participant was prepared for the EEG measurements, which took approximately 30 min. The participant was then seated in a dimly lit, sound‐attenuating cabin, approximately 60 cm from a computer screen. The experiment was carried out with OpenSesame software, version 3 (Mathôt et al. 2012) and started with 4 practice trials to get familiar with the procedure, which showed IAPS images that were not part of the experiment (see Table A1). The actual experiment followed, showing 150 images in random order, with a short break after 75 trials. The participant provided subjective valence and arousal ratings following each image. The experiment took about 20 min on average.

### 2.5 Physiological Measurements

EEG was recorded with 32 Ag‐AgCl ActiveTwo electrodes (BioSemi, Amsterdam, The Netherlands) in an extended 10/20 layout (Chatrian et al. 1985) with a sampling rate of 512 Hz. Two electrodes were applied to the mastoids for offline re‐referencing. Electrodes for the detection of eye blinks and eye movements were applied above and below the right eye and next to the lateral canthi of the eyes.

### 2.6 Preprocessing

The raw EEG data were preprocessed in BrainVision Analyzer 2.1.2 (Brain Products GmbH). First, channels with overall poor signal quality were reconstructed by fourth‐order spherical splines interpolation. The number of reconstructed channels was limited to three (< 10%) per participant. On average, 0.6 channels were reconstructed per participant. The data were then re‐referenced to the mean of the mastoid channels. Slow drift and high‐frequency noise were filtered out using zero‐phase shift Butterworth filters (0.01 Hz high‐pass and 70 Hz low‐pass, −24 dB/octave roll‐off). A notch filter was applied at 50 Hz to remove power line interference. The signal was then segmented into epochs from 1000 ms before until 2500 ms after stimulus onset. Next, artifacts caused by eye blinks and eye movements were corrected using an automated ocular correction independent component analysis (ICA). Trials with remaining artifacts were then automatically detected and rejected according to the criteria of exceeding a voltage step of 50 μV/ms or exceeding the max/min difference of 200 μV within the 3500 ms duration of a trial segment. A predefined criterion of having no more than half of the trials rejected in each condition was used to exclude poor‐quality datasets from further analysis. On average, there were 42.6 positive, 42.7 negative, and 42.8 neutral trials left after artifact rejection in the remaining data.

Further processing steps and analyses were performed in MATLAB (v. R2023b, MathWorks Inc.) using the FieldTrip Toolbox (Oostenveld et al. 2011). The data were low‐pass filtered at 30 Hz prior to ERP analysis. Baseline correction was applied using the 200‐ms interval leading up to stimulus onset as the baseline interval.

### 2.7 Statistical Analysis

#### 2.7.1 Subjective Ratings

To assess whether the predefined valence conditions were experienced by the participants conforming to the IAPS normed valence ratings: clearly distinct in valence, yet largely overlapping in arousal ratings, we analyzed the subjective valence and arousal ratings through repeated measures ANOVAs. Greenhouse–Geisser corrections were applied. Effect sizes are provided as partial eta squared (*η* ^2^). Significant differences were followed up with simple effects tests, for which Cohen's *d* effect sizes are reported.

Affective salience ratings were calculated from the subjective valence ratings for each image for each participant, as the distance from the middle of the rating range: abs(valence rating −5). Differences in these salience ratings between the valence conditions were analyzed and reported in the same way as the valence and arousal ratings.

#### 2.7.2 Event‐Related Potentials

Comparisons between categories were performed pairwise: positive vs. negative, positive vs. neutral, and negative vs. neutral. Differences between conditions in ERP amplitudes were analyzed with nonparametric cluster‐based random permutation analysis (Maris and Oostenveld 2007) on the 2500‐ms interval following stimulus onset.

This method does not require splitting the interval into a priori specified segments that would inevitably be misaligned to some degree with the effects that are present in the data. Instead, the statistical significance of the pairwise difference between conditions is determined per time point, per channel, through Monte Carlo estimates of random permutation tests. First, the average amplitude of the EEG signal over all trials in a condition is calculated for each participant, for each condition. Next, for each time point and for each channel, the permutation distribution of the amplitudes is calculated for each condition, over participants. For each of these, a p‐value for the null hypothesis is determined with the formula of the paired‐sample t‐test. Samples with statistically significant differences between conditions are then clustered on the basis of temporal and spatial adjacency. Multiple comparison correction is applied on the cluster level; see Maris and Oostenveld (2007) for a detailed description.

For the sake of readability, only significant *p*‐values are provided. Cohen's *d* effect sizes were calculated for the difference in ERP amplitude, averaged over those channels/time points that were part of the cluster.

#### 2.7.3 Multilevel Analyses

To facilitate making informed inferences about the relationships between the ERP responses and the experiences of valence, arousal, and affective salience, we performed multilevel analyses. Average ERP amplitudes for each of the two latency intervals (LPP: 500–1300 ms, long‐latency: 1500–2500 ms) were predicted by each of the three subjective rating scales (valence, arousal, affective salience), resulting in six separate models. The three valence conditions were used as the repeated measures variable. To take interindividual differences into account, the participant was included as a grouping variable. ERP amplitudes were first aggregated to a single number per condition per participant by averaging over all available trials within a condition, over the channels having the most pronounced amplitudes in both intervals (Cz, CP1, CP2, Pz), and over all samples within the respective latency interval. Subjective ratings were averaged per participant, over all trials within each condition for each of the three rating scales. Linear mixed‐effects models were then fitted, predicting the average ERP amplitude using a fixed intercept and fixed effect of the ratings (slope), and a random intercept and effect of the ratings (slope) that were allowed to vary between participants, as in (1). Maximum likelihood estimation was used as a method to estimate the parameters of the linear mixed‐effects model for each of the six ERP interval × subjective rating pairs separately.

(1) $$\mathrm{ERP}\sim 1+\text{rating}+\left(1+\text{rating}\;|\text{participant}\right)$$

## 3 Results

### 3.1 Subjective Ratings

**FIGURE 2:**
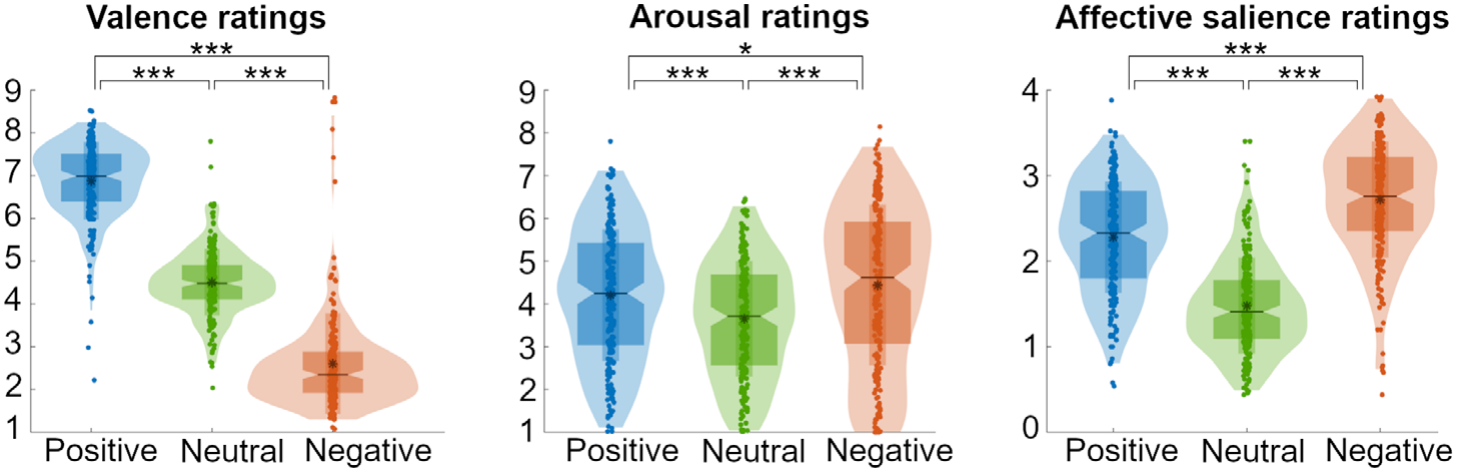
Distribution of the valence, arousal, and affective salience ratings per category. The mean subjective valence (left panel), arousal (middle panel), and affective salience ratings (right panel) for each of the positive, neutral, and negative categories are shown. Dots represent the average ratings over the images within a category, per participant. The violin‐shaped areas represent the distribution density of the ratings. Horizontal black lines represent the median, and asterisks represent the means. Dark shapes around the median show the first to third quartile. Lighter rectangles within indicate one standard deviation below to one standard deviation above the mean. Violin plots were created with al_goodplot by Legouhy 2023. Asterisks indicate significant differences between rating categories (*p < 0.05, ***p < 0.001).

Subjective valence ratings (Figure 2, left panel) differed between the predetermined valence conditions, *F*(1.134, 252.9) = 1096.4, *p* < 0.001, *partial η* ^2^ = 0.83. Valence ratings of images in the positive category (*M* = 6.87, SD = 0.91) were higher (*t*(223) = 28.99, *p* < 0.001, *d* = 2.8) than those in the neutral category (*M* = 4.51, SD = 0.78), which in turn were higher, *t*(223) = 34.50, *p* < 0.001, *d* = 1.75, than those in the negative category (*M* = 2.60, SD = 1.18). It naturally follows that images in the positive category were rated more positively than those in the negative category, *t*(223) = 34.48, *p* < 0.001, *d* = 4.09. The large effect sizes of these results show that the valence categories were clearly distinct in subjective valence ratings, as intended.

The distribution of subjective arousal ratings (Figure 2, middle panel) showed considerable overlap between positive (*M* = 4.20, SD = 1.54), neutral (*M* = 3.66, SD = 1.35), and negative categories (*M* = 4.44, SD = 1.87), as intended. However, ratings did differ between the predetermined valence conditions, *F*(1.36, 303.3) = 37.9, *p* < 0.001, *partial η* ^2^ = 0.15. Arousal ratings were significantly higher for both the positive, *t*(223) = 8.05, *p* < 0.001, *d* = 0.37, and the negative, *t*(223) = 9.54, *p* < 0.001, *d* = 0.45, compared to the neutral category. They were also significantly higher for the negative compared to the positive category, *t*(223) = 2.01, *p* = 0.045, *d* = 0.14. Effect sizes of these differences in arousal between categories were small for the positive vs. negative comparison and moderate for the other comparisons.

Subjective ratings of affective salience (Figure 2, right panel) differed between the predetermined valence conditions, *F*(1.82, 405.16) = 496.8, *p* < 0.001, *partial η* ^2^ = 0.69. Images in the positive valence condition (*M* = 2.28, SD = 0.65) were rated higher on affective salience (*t*(223) = 19.17, *p* < 0.001, *d* = 1.3) than neutral images (*M* = 1.48, SD = 0.56). Images in the negative valence condition (*M* = 2.72, SD = 0.68) were also rated higher on affective salience (*t*(223) = 37.42, *p* < 0.001, *d* = 1.95) than neutral images. Images in the negative category were rated as more salient than those in the positive category, *t*(223) = 10.01, *p* < 0.001, *d* = 0.66. The large effect sizes for the positive and negative compared to the neutral images show that these were much more salient, as intended. However, the medium effects size for the negative compared to the positive images indicate greater affective salience for the negative image category, while these were expected to be approximately equal.

Pearson correlations indicated a high level of agreement between the subjective ratings given by our participants and normative ratings as listed in Lang et al. (2008) for both valence (*r* = 0.96, *p* < 0.001) and arousal (*r* = 0.82, *p* < 0.001).

### 3.2 Event‐Related Potentials

**FIGURE 3:**
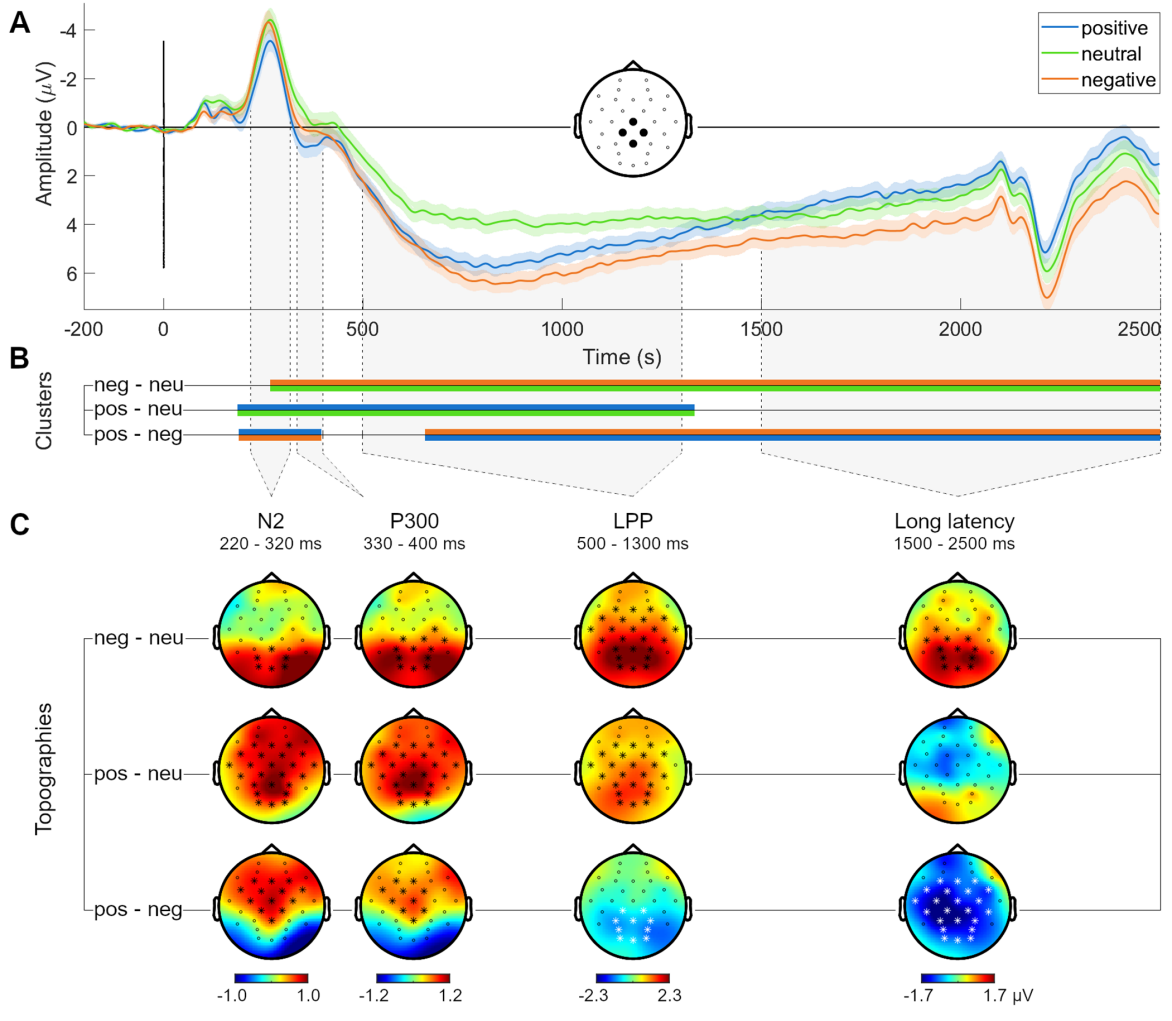
ERPs to the valence conditions with cluster intervals and topographies of pairwise differences. (A) The averaged EEG signals to the positive (blue), neutral (green), and negative (orange) valenced images. The signal is averaged across the central‐parietal electrodes (Cz, CP1, CP2, Pz) where the differences are most pronounced. Shaded areas around the EEG represent the standard error of the mean. The direction of the y‐axis is inverted (i.e., negative is up) to conform to existing literature. The peak starting at 2200 ms is the offset potential, caused by the image being removed at 2000 ms. (B) Indication of the latency intervals of the clusters that constitute pairwise differences in amplitude. (C) Topographies of the pairwise difference in EEG amplitude, averaged over the latency intervals that correspond to the ERP components that fell within the latency interval of one or more clusters. Intervals were chosen to fit both the current data and the literature. Circles represent electrode positions; asterisks indicate that an electrode was part of a cluster of significant differences.

Visual inspection of the data (Figure 3A) suggests a clear and long‐lasting salience effect in the LPP from around 500 to 1300 ms, with larger amplitudes for the positive and negative valence conditions compared to the neutral valence condition. Amplitudes for the negative valence condition were also (to a lesser extent) larger than those for the positive condition, which is in line with differences in subjective ratings on affective salience (and of arousal) that were also somewhat greater for the negative compared to the positive valence condition.

Between 1300 and 1500 ms, amplitudes of the positive and negative valence conditions started to diverge (Figure 3A). From around 1500 ms, amplitudes for the neutral valence condition were between those of the positive and negative conditions. ERPs are ordered according to valence from 1500 ms onward, suggestive of a valence effect that appears to extend beyond the end of the analysis interval at 2500 ms.

#### 3.2.1 Clusters of Significant Differences

Pairwise cluster‐based permutation tests on the ERP amplitudes in the positive, neutral, and negative valence categories revealed a cluster of larger ERP amplitudes to the positive compared to the neutral valence category, which extended from 186 ms until 1332 ms (*p* < 0.001, *d* = 0.42, Figure 3B). A cluster of larger ERP amplitudes in the negative compared to the neutral valence condition extended from 268 ms until the end of the analysis interval at 2500 ms (*p* < 0.001, *d* = 0.51, Figure 3B).

The comparison of the negative against the positive valence condition resulted in two clusters of significant differences. From 188 to 396 ms, amplitudes were larger for the positive compared to the negative valence condition (*p* = 0.019, *d* = 0.30). This was followed by a long‐lasting greater deflection for the negative valence compared to the positive valence condition in the positive‐going LPP and beyond (*p* < 0.001, *d* = 0.32), from 656 to 2500 ms (Figure 3B).

#### 3.2.2 ERP Components

*N2*. During the N2 interval [220–320 ms], clusters indicated that amplitudes were larger for the positive compared to both the negative and the neutral valence conditions (Figure 3B). This difference was most pronounced in central areas (Figure 3C). A cluster of larger amplitudes for the negative compared to the neutral condition, at occipital areas, partially overlapped with the N2 interval.

*P300*. Effects observed during the subsequent P300 interval [330–400 ms] were very similar to those in the N2 interval in terms of both amplitude and topography (Figure 3B,C). The cluster of larger amplitudes for the negative compared to the neutral condition fully overlapped with the P300 interval, encompassing parietal and parietal‐central areas in addition to occipital areas.

*LPP*. Amplitudes of the LPP [500–1300 ms] were larger for the positive and negative valence conditions compared to the neutral one (Figure 3B). These differences were most pronounced at central and parietal areas (Figure 3C). This pattern of amplitude differences, the latency interval, and topography are consistent with an affective salience effect, as expected. For part of the interval, amplitudes were larger for the negative compared to the positive valence condition at occipital and parietal areas (Figure 3C).

*Long‐latency interval*. Although cluster analysis results show that amplitudes were larger for the negative valence condition compared to the positive valence condition from around 650 ms, the relation to amplitudes for the neutral condition suggests a qualitative switch from an affective salience effect to a valence effect around 1500 ms. At longer latencies, amplitudes for the neutral condition were in between those for the negative and positive valence conditions. Amplitudes were hence in an order that corresponds to valence (Figure 3B) in the [1500–2500 ms] interval, indicative of a valence effect. However, the direction of this effect was opposite from what we had predicted based on the attractive faces study (Revers et al. 2023), where attractive faces (positive valence) had larger amplitudes in the long‐latency interval.

Amplitudes were larger for the negatively valenced images compared to the positively valenced images in central, parietal, and frontal‐central areas (Figure 3C) and larger for the negatively valenced images compared to the neutral images in parietal and occipital areas (Figure 3C).

### 3.3 Multilevel Analyses

To guide inferences on the relationship between ERP responses in the LPP (500–1300 ms) and long‐latency interval (1500–2500 ms) with subjective ratings of valence, arousal, and affective salience, we performed multilevel analyses to fit each of the six models (2 ERP intervals x 3 ratings). The intercept and slope were allowed to vary between participants to take individual differences into account.

LPP amplitudes were significantly associated with both the arousal and affective salience ratings (*p* < 0.01; see Table 2), but not with the valence ratings (*p* = 0.07). Since arousal and affective salience overlap to some degree, we followed up on these results by taking the model that predicted LPP amplitude from one rating type and adding the other rating type as a predictor (as fixed and random intercept and slope).

**TABLE 2:** Slope (and *p* value) for each of the multilevel models.

	Valence	Arousal	Affective salience
LPP	−0.09 (*p* = 0.07)	0.30 (*p* = 0.004)**	1.02 (*p* < 0.001)***
Long‐latency	−0.26 (*p* < 0.001)***	−0.14 (*p* = 0.34)	0.07 (*p* = 0.75)

*Note:* Asterisks indicate the level of significance (*p* < 0.05*, 0.01**, 0.001***).

A likelihood ratio test indicated that adding arousal ratings to the model that already had affective salience as predictor did not improve the prediction of the model (*p* = 0.64). However, adding affective salience ratings to the model that predicted LPP amplitudes from arousal ratings did improve the model significantly (*p* < 0.001). Taken together, these two results suggest that arousal ratings did not have predictive value for LPP amplitudes that is not already captured by the affective salience ratings.

Amplitudes in the long‐latency interval were significantly associated with the valence ratings, but not with arousal and affective salience ratings (Table 2).

Based on these results, we infer that in the current study, ERP responses in the LPP interval were neural representations of experiences of affective salience, and ERP responses in the long‐latency interval were neural representations of experiences of valence.

## 4 Discussion

The aim of the present study was to verify the existence of robust and replicable valence effects in ERPs at very long latencies (> 1000 ms), following up on an initial report by Revers et al. (2023). 224 participants viewed IAPS images in three valence categories (positive, neutral, negative) while their EEG was measured. ERP amplitudes were larger for both the positive and negative valence categories compared to the neutral category in a (relatively) early time interval of 500–1000 ms poststimulus. This affective salience effect was followed by a valence effect that started around 1500 ms and extended beyond the end of the current analysis window (2500 ms poststimulus), at frontal‐central, central, and parietal regions. While the valence effect itself was hypothesized, it was in the opposite direction from what we had expected. Additionally, differential effects in the N2 and P300 components were found, though these were not clearly interpretable.

### 4.1 Affective Salience Effect in the LPP Interval

The affective salience effect in the LPP interval has been reported frequently and consistently in the affective neuroscience literature (Hajcak et al. 2011; Olofsson et al. 2008). Considering the relatively large sample of participants and the relatively large number of stimuli in the current study, the clear and long‐lasting ERP differences between the positive and negative compared to the neutral valence condition in the current data add to the robustness of the claim that the LPP is sensitive to affective salience.

LPP amplitudes were also larger for the negative valence condition compared to the positive valence condition, in line with the greater subjective ratings of affective salience for the negative compared to the positive condition.

While the pattern in ERP results matches that in subjective ratings of both arousal and affective salience, affective salience ratings were a better predictor of ERP amplitude. Arousal does not add to this prediction beyond affective salience. The relationship between affective salience and arousal is complex and a matter of debate (Haj‐Ali et al. 2020; Kron et al. 2015). It may depend on the person, circumstances (Kuppens et al. 2013), and cultures (Yik et al. 2023). In the current results, differences in LPP amplitude are best predicted by differences in affective salience. However, we have purposefully selected our stimuli in such a way that arousal ratings were similar across conditions with a high degree of overlap between conditions. The goal of this selection was to minimize the arousal effect. As a result, similar analyses on more general stimulus sets would be needed to generalize our conclusions.

### 4.2 Valence Effect in the Long‐Latency Interval

The ERPs of the current study showed a valence effect that started around 1500 ms and lasted beyond the end of the analysis interval at 2500 ms. While amplitudes were already larger for the negative compared to the positive valence condition during the LPP interval, a qualitative switch from an affective salience effect to a valence effect is observable at around 1500 ms, which was confirmed by the multilevel analysis. ERP amplitudes diverged between the positive and negative valence conditions from around 1300 ms onward, and amplitudes for the neutral condition were in between those from 1500 ms, resulting in a clear ordering according to valence (negative > neutral > positive; Figure 3B).

Latencies above 1000 ms are not typically explored in affective neuroscience, with few exceptions (e.g., Cuthbert et al. 2000; Revers et al. 2023; Yen et al. 2010). Hence, valence effects in ERPs have long been overlooked because of the limits that have been imposed on the analysis time window. However, our present data confirm the existence of a clear, robust, and substantively sized valence effect (Cohen's *d* values of 0.3–0.5) at the unusually long latencies of 1500‐2500 ms, as previously reported by Revers et al. (2023) for faces.

However, in that study, images of attractive, unattractive, and intermediate attractive faces were used to elicit positive, negative, and neutral valence, respectively. Those face images had been carefully chosen so that they were very similar in all respects except attractiveness. While that allows for strong inferences about the cause of the effects, it restricts generalizing those inferences to the general concept of valence. Additionally, face (attractiveness) processing is a swift and automatic, evolution‐based response (Olson and Marshuetz 2005) in brain circuits that are dedicated to face processing (Kanwisher and Yovel 2006), further hindering generalization to more general affect processing. The current study used complex images of sceneries from the widely used IAPS database (Lang et al. 2008), which is generally recognized to elicit valence and arousal effects. Therefore, the observed valence effect in this study suggests that very long‐latency valence effects reflect more domain‐general valence processing.

It should be noted that, while both the present study and the facial attractiveness study showed a parametric effect of valence in ERP amplitudes—ordered according to valence conditions in the long‐latency interval—a notable difference was observed between the two studies. The order of the effect was in opposite directions. Here, negative valence was associated with larger amplitudes than positive valence, while Revers et al. (2023) reported the opposite pattern.

The two results could perhaps be reconciled within a theoretical framework that focuses on the importance of maintaining allostasis, which is the efficient control of resources for all physiological systems that are needed for growth, survival, and reproduction (Barrett 2017b). Some events have greater potential impact on allostasis than others, requiring further processing, while inconsequential stimuli can be safely ignored (Barrett 2017b). Further processing could involve, for example, anticipating consequences, determining the best course of action, and encoding the event in memory. This second stage of stimulus processing might be dependent on the interplay between the allostatic situation of the observer and the valence of the stimulus.

We have not measured nor asked the perceived level of impact on allostasis, so this is an untested hypothesis at this point. However, it seems plausible considering the differential impact that the two sets of stimuli might have had on allostasis. First, a highly attractive face (which is associated with positive valence) is an indicator of good health (Jones et al. 2001; Thornhill and Gangestad 1993) and reproductive potency (Lie et al. 2008; Soler et al. 2003) which is relevant for the reproductive aspect of allostasis and warrants further, more detailed processing. Noteworthy here is that the late valence effect of facial attractiveness was observed only for faces of the preferred gender, not for the dispreferred gender (Revers et al. 2023), which suggests that allostatic relevance is a determining factor for the differential neural activity in the long‐latency interval that makes up the valence effect.

In contrast, in the current study, the negatively valenced IAPS images could presumably be most relevant for the survival aspect of allostasis. These images depict threats, mutilation, disease, accidents, etc. The high allostatic relevance presumably prompts further processing involving the anticipation of consequences, determining the best course of action, and encoding the event in memory. The positive images, on the other hand, require little further processing as their content has little impact on allostasis. In sum, both positively valenced facial attractiveness stimuli from the preferred gender and negatively valenced IAPS stimuli might evoke the greatest need for maintaining allostasis, and thus elicit greater neural processing subsequent to the LPP. If so, then the opposite‐direction valence effects that are observed in both studies in the long‐latency interval might be more closely related to allostatic relevance than to valence direction per se. This allostasis account of valence might also explain why Cuthbert et al. (2000) found no valence effect at long latencies. In their study, ERP amplitudes at long latencies were higher for the positive and negative images compared to neutral images. Their positive images included erotic content (Yen et al. 2010), which made them relevant for the procreation aspect of allostasis. The negative images included mutilation, attack, and an angry face; hence, these were also relevant to allostasis (for the survival aspect). Consequently, long‐latency amplitudes were increased in both the positive and the negative valence condition. It seems therefore relevant that future studies manipulate (or control) both valence and allostatic relevance to further explore the processing of valence and the possible role of allostatic relevance.

### 4.3 Emotion Theories

The current clear and long‐lasting affective salience and valence effects are in line with two‐stage theories of emotions. The two effects are clearly distinct in time, with a sequential order that suggests that affective salience is processed first, followed by the processing of valence. Such sequential processing is in agreement with emotion theories that assume distinct stages in emotion processing (MacCormack and Lindquist 2017) like the two‐factor theory of emotion (Schachter and Singer 1962), the James‐Lange theory (Dewey 1894; James 1884; Lange 1885), and the theory of constructed emotions (Barrett 2017a).

An event is first evaluated on its potential impact on allostasis. Internal sensations from the body are sent to the brain to provide information about the status of the body (e.g., its resources). Interoception, the processing of these signals, takes place in the anterior insula, a part of the affective salience network (Craig 2009). Activity in this area correlates with amplitudes of the LPP (Liu et al. 2012). This suggests that the processing of interoceptive signals and evaluation of allostatic impact takes place during the LPP interval. Affective (positive and negative valenced) stimuli have greater potential consequences for allostasis, and thus are worth the energy expenditure of further neural processing. The neutral stimuli, on the other hand, are inconsequential and no more energy should be spent on these because the brain must be energy efficient (Padamsey and Rochefort 2023). This greater neural processing of affective events compared to neutral ones would be observable as the affective salience effect in the LPP, and as differential BOLD responses in the affective salience network (Liu et al. 2012; Schweizer et al. 2019).

Next, integration of the content of the stimulus, the current state of the body, and episodic memory of similar events determines whether the event (the stimulus) is relevant for allostasis and requires further processing in greater detail to ensure a desired outcome, while others are safely ignored (Barrett 2017b). This could be the affective valence effect that we observed in the long latencies that follow the LPP component.

### 4.4 Other ERP Components

In addition to the main findings of the present study, differences between valence categories were also found for latencies corresponding with the well‐established N2 and P300 components, but the nature of these differences is difficult to interpret.

Both the negative and neutral valence images elicited larger deflections of the N2 component compared to the positive images. This pattern was partly reversed in the interval of the positively going P300, with larger deflections for positive images compared to both negative and neutral images, and larger deflections for the negative images compared to the neutral images. Earlier studies have reported larger N2 and P300 amplitudes for positively and negatively valenced stimuli compared to neutral stimuli (Hajcak et al. 2011). However, in the current findings, neither of these effects shows an ordered pattern that clearly matches an affective salience (positive/negative > neutral) or valence effect (positive > neutral > negative, or vice versa), making them difficult to interpret in the context of our affective manipulation.

In the current results, ERP responses to positive and negative images started to deviate from those to neutral images at early latencies (186 and 268 ms, respectively) that very closely matched those reported by Bo et al. (2022) who used decoding techniques to explore the temporal dynamics of affective scene processing. It is possible that these early effects reflect fast emotion processing that does not require consciousness, as suggested by LeDoux (1996) and Öhman (1992). However, the quick processing of threat, especially evolutionarily relevant threat, would be expected to show the greatest neural activity (LeDoux 2012; Ohman 2005), which is not what we see in the current N2 and P300 effects.

While the images in the positive valence were somewhat brighter than those in the neutral and negative valence conditions, this is unlikely to have had an impact on the ERP differences that we found. Luminance has been reported to affect early ERPs like the latency of the occipital N1 component (Park et al. 2013) but is not known to affect later components at central areas.

### 4.5 Conclusion

In this study, we have established the presence of a robust and sizeable valence effect in the long‐latency interval of the ERP. Additionally, we have replicated the affective salience effect in the LPP component, solidifying the general consensus that the LPP is sensitive to affective salience. We further found preliminary evidence that valence processing interacts with allostatic relevance. Valenced events are presumably processed further by the brain if the content of the event is personally relevant, e.g., for survival, growth, or procreation. Future empirical studies should test this hypothesis. The current results provide robust evidence supporting the notion that experiencing affective valence is a two‐stage sequential process.

## Author Contributions

**Hans Revers:** conceptualization, data curation, formal analysis, investigation, methodology, project administration, resources, software, visualization, writing – original draft. **Jeroen J. Stekelenburg:** conceptualization, data curation, investigation, methodology, project administration, resources, validation, writing – review and editing. **Jean Vroomen:** writing – review and editing. **Katrijn Van Deun:** writing – review and editing. **Marcel Bastiaansen:** conceptualization, methodology, supervision, validation, writing – review and editing.

## Conflicts of Interest

The authors declare no conflicts of interest.

## Data Availability

The data and code supporting the findings of this study are available on DataVerseNL: https://dataverse.nl/dataverse/cognitive_neuropsychology.

## Appendix A

**TABLE A1:** Overview of all 150 images that were shown in the experiment, 50 images in each of the positive, neutral, and negative categories.

Negative			Neutral			Positive		
IAPS	Valence	Arousal	IAPS	Valence	Arousal	IAPS	Valence	Arousal
2205	1.95	4.53	1022	4.26	6.02	1440	8.19	4.61
2276	2.67	4.63	1030	4.30	5.46	1441	7.97	3.94
2301	2.78	4.57	1303	4.68	5.7	1460	8.21	4.31
2352.2	2.09	6.25	1310	4.60	6.00	1463	7.45	4.79
2375.1	2.20	4.88	1321	4.32	6.64	1500	7.24	4.12
2455	2.96	4.46	1350	5.25	4.37	1630	7.26	4.45
2456	2.84	4.55	1390	4.5	5.29	1750	8.28	4.10
2683	2.62	6.21	1616	5.21	3.95	2080	8.09	4.70
2750	2.56	4.31	1645	4.99	5.14	2091	7.68	4.51
2900.1	2.56	4.61	1726	4.79	6.23	2154	8.03	4.48
3005.1	1.63	6.20	1820	5.35	5.67	2216	7.57	5.83
3019	2.99	6.30	1908	5.28	4.88	2222	7.11	4.08
3060	1.79	7.12	2458	4.69	5.28	2224	7.24	4.85
3195	2.06	6.36	2487	5.20	4.05	2306	7.08	4.46
3300	2.74	4.55	2579	5.53	3.85	2314	7.55	4.00
6230	2.37	7.35	2690	4.78	4.02	2530	7.80	3.99
6260	2.44	6.93	2702	5.21	3.92	4608	7.07	6.47
6415	2.21	6.20	2704	4.85	5.30	4660	7.40	6.58
6550	2.73	7.09	2780	4.77	4.86	5210	8.03	4.60
6821	2.38	6.29	3210	4.49	5.39	5450	7.01	5.84
6834	2.91	6.28	4631	5.36	5.19	5460	7.33	5.87
9000	2.55	4.06	5920	5.16	6.23	5470	7.35	6.02
9041	2.98	4.64	6570.2	4.86	3.85	5594	7.39	4.15
9050	2.43	6.36	6837	4.25	4.50	5621	7.57	6.99
9183	1.69	6.58	6900	4.76	5.64	5629	7.03	6.55
9187	1.81	6.45	6910	5.31	5.62	5781	7.13	3.82
9220	2.06	4.00	7018	4.81	3.91	5814	7.15	4.82
9265	2.6	4.34	7033	5.40	3.99	5831	7.63	4.43
9280	2.8	4.26	7044	4.69	3.94	5836	7.25	4.28
9290	2.88	4.40	7058	5.29	3.98	7200	7.63	4.87
9291	2.93	4.38	7077	5.12	4.61	7270	7.53	5.76
9300	2.26	6.00	7081	5.36	3.96	7350	7.10	4.97
9321	2.81	6.24	7242	5.28	3.83	7405	7.38	6.28
9330	2.89	4.35	7497	5.19	4.97	7470	7.08	4.64
9331	2.87	3.85	7504	5.67	4.25	7502	7.75	5.91
9342	2.85	4.49	7506	5.34	4.25	8030	7.33	7.35
9414	2.06	6.49	7560	4.47	5.24	8163	7.14	6.53
9415	2.82	4.91	7632	5.22	4.78	8180	7.12	6.59
9432	2.56	4.92	7640	5.00	6.03	8186	7.01	6.84
9561	2.68	4.79	8060	5.36	5.31	8190	8.10	6.28
9600	2.48	6.46	8065	5.25	5.71	8200	7.54	6.35
9630	2.96	6.06	8160	5.07	6.97	8210	7.53	5.94
9635.1	1.90	6.54	8192	5.52	6.03	8300	7.02	6.14
9800	2.04	6.05	8325	5.63	4.47	8370	7.77	6.73
9810	2.09	6.62	8475	4.85	6.52	8380	7.56	5.74
9830	2.54	4.86	9150	4.54	5.31	8492	7.21	7.31
9831	2.95	4.61	9401	4.53	3.88	8496	7.58	5.79
9832	2.94	4.46	9411	4.63	5.37	8497	7.26	4.19
9908	2.34	6.63	9468	4.67	4.68	8499	7.63	6.07
9921	2.04	6.52	9913	4.38	4.42	8501	7.91	6.44

*Note:* IAPS image numbers, normative valence, and arousal rating as reported in the technical manual of the International Affective Picture System (IAPS; Lang et al. 2008).
